# A Nomogram for Predicting the Residual Back Pain after Percutaneous Vertebroplasty for Osteoporotic Vertebral Compression Fractures

**DOI:** 10.1155/2021/3624614

**Published:** 2021-11-01

**Authors:** Qiujiang Li, Lin Shi, Yinbin Wang, Tao Guan, Xiaocheng Jiang, Donggeng Guo, Jinhan Lv, Lijun Cai

**Affiliations:** ^1^Graduate School of Ningxia Medical University, Yinchuan, Ningxia, China; ^2^Department of Orthopedics, People's Hospital of Ningxia Hui Autonomous Region, Yinchuan, Ningxia, China; ^3^Traditional Chinese Medicine Hospital Dianjiang Chongqing, Chongqing, China

## Abstract

**Objective:**

Current findings suggest that percutaneous vertebroplasty (PVP) is a suitable therapeutic approach for osteoporotic vertebral compression fractures (OVCFs). However, a significant minority of patients still experience residual back pain after PVP. The present retrospective study was designed to determine the risk factors for residual back pain after PVP and provides a nomogram for predicting the residual back pain after PVP.

**Methods:**

We retrospectively reviewed the medical records of patients with single-segment OVCFs who underwent bilateral percutaneous vertebroplasty. Patients were divided into group N and group R according to the postoperative VAS score. Group R is described as the VAS score of residual back pain ≥ 4. Pre- and postoperative factors that may affect back pain relief were evaluated between two groups. Univariate and multivariate logistic regression analysis were performed to identify risk factors affecting residual back pain after PVP. We provided a nomogram for predicting the residual back pain and used the receiver operating characteristic curve (ROC), concordance index (C-index), calibration curve, and decision curve analyses (DCA) to evaluate the prognostic performance.

**Results:**

Among 268 patients treated with PVP, 37 (13.81%) patients were classified postoperative residual back pain. The results of the multivariate logistical regression analysis showed that the presence of an intravertebral vacuum cleft (IVC) (OR 3.790, *P*=0.026), posterior fascia oedema (OR 3.965, *P*=0.022), severe paraspinal muscle degeneration (OR 5.804, *P*=0.01; OR 13.767, *P* < 0.001), and blocky cement distribution (OR 2.225, *P*=0.041) were independent risk factors for residual back pain after PVP. The AUC value was 0.780, suggesting that the predictive ability was excellent. The prediction nomogram presented good discrimination, with a C-index of 0.774 (0.696∼0.852) and was validated to be 0.752 through bootstrapping validation. The calibration curve of the nomogram demonstrated a good consistency between the probabilities predicted by the nomogram and the actual probabilities. The nomogram showed net benefits in the range from 0.06 to 0.66 in DCA.

**Conclusions:**

The presence of IVC, posterior fascia oedema, blocky cement distribution, and severe paraspinal muscle degeneration were significant risk factors for residual back pain after PVP for OVCFs. Patients with OVCFs after PVP who have these risk factors should be carefully monitored for the possible development of residual back pain. We provide a nomogram for predicting the residual back pain after PVP.

## 1. Introduction

With the aging of the social population, incidence of osteoporosis is constantly increasing, seriously affecting life quality of elderly patients [1]. Osteoporotic vertebral compression fractures (OVCFs), one of the most common complications of osteoporosis, often occur in low-energy damage or the absence of a clear trauma history, which primarily results in persistent back pain, local vertebral kyphosis, a reduced quality of life as well as increased mortality [2,3]. Percutaneous vertebroplasty can provide instant pain relief and stabilize the fractured vertebral body through the minimally invasive injection of polymethyl methacrylate (PMMA) bone cement, which has been widely used in OVCFs treatment [4].

However, residual back pain still exists in a proportion of patients after percutaneous vertebroplasty (PVP), and it even affects daily life due to moderate or severe back pain, which is of great concern [5]. It has been investigated that the percentage of unrelieved back pain after PVP is about 5% to 20% [6–8]. Previously, a few studies have reported the possible influencing factors affecting residual back pain after PVP, including bone density, bone cement volume, bone cement distribution, and thoracolumbar dorsal fascia injury [7–9]. However, there are fewer related studies and the conclusions are somewhat controversial. In contrast, paravertebral muscle degeneration was confirmed to be an important risk factor for the development of chronic low back pain [10,11]. We speculate that paraspinal muscle degeneration has a relationship with residual back pain in patients after PVP. Therefore, the present study was conducted to provide a comprehensive analysis of possible risk factors and a nomogram for predicting the residual back pain, which could help to improve the clinical prognosis by early intervention in patients.

## 2. Methods

### 2.1. Participant Population

**Figure 1:**
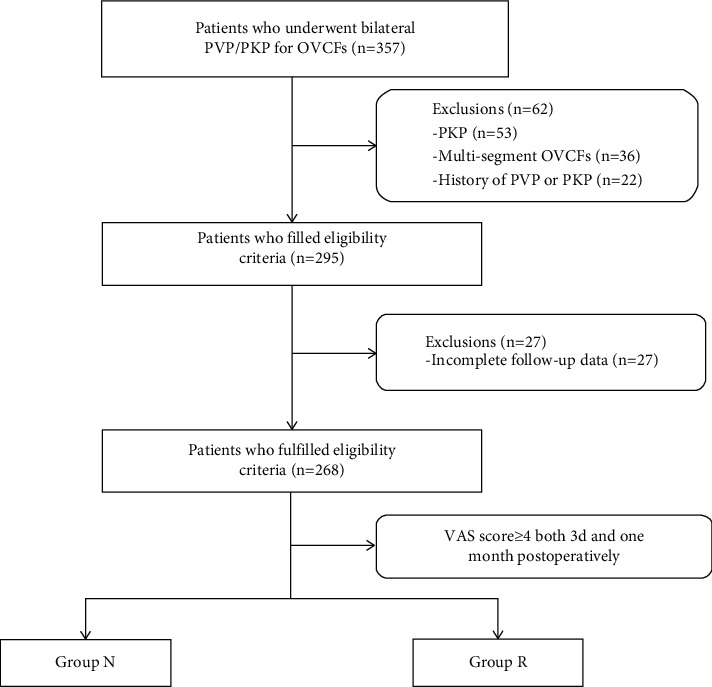
Patient flowchart.

The clinical data of patients with single-segment OVCFs who underwent bilateral percutaneous vertebroplasty from January 2017 to April 2019 were retrospectively reviewed. The inclusion criteria are as follows: (1) patients having obvious thoracolumbar and back pain and limited physical activity, especially in cases of turning over or getting up. (2) T score ≤ −2.5 at spine/hip with dual energy X-ray absorptiometry (DXA). (3) The signal change of the lumbar fracture of lumbar magnetic resonance imaging (MRI) suggesting a hyperintense T2 signal and a hypointense T1 signal or a whole-body bone scan performed an active bone metabolism. Exclusion criteria are as follows: (1) patients with OVCFs caused by tumor, infection, or tuberculosis. (2) Patients having coagulation dysfunction, combined systemic disease, and inability to tolerate the procedure. (3) Systemic or local infection. (4) Spinal cord compression and obvious neural symptoms such as numbness and/or muscle weakness. (5) Incomplete follow-up data. From January 2017 to April 2019, a total of 357 patients in our institution were diagnosed with OVCFs and underwent bilateral PVP/PKP. Of these 357 patients, 89 who did not meet the inclusion criteria or meet any of the exclusion criteria were excluded. Finally, our study screened a total of 268 patients who met the criteria, including 53 males and 215 females.  Figure 1 shows the patient flowchart. The present study was approved by the medical ethics committee of the Peoples Hospital of Ningxia Hui Autonomous Region. All included patients signed an informed consent.

### 2.2. Surgical Method

The patient was placed in the prone position, the abdomen was vacated, and the fractured vertebrae were located under C-arm fluoroscopic guidance. The puncture needle was inserted into the vertebral body via bilateral arch pathways. The tip of the puncture needle was located in the anterior middle third of the vertebral body on the lateral view, and the anterior-posterior view was located between the inner edge of the ipsilateral pedicle and the vertebral body midline. The working channel is established and the high-viscosity cement is slowly injected under C-arm fluoroscopy until the bone cement approaches the posterior wall of the vertebral body where leakage may occur, and the working channel is slowly withdrawn after the cement has hardened. The whole procedure was done with the assistance of C-arm fluoroscopy.

### 2.3. Postoperative Management

All patients were given oral calcium and vitamin D postoperatively and an intravenous infusion of zoledronic acid (Aclasta, 100 ml/5 mg) once a year thereafter for 3 years. Patients were reviewed on the postoperative 24 h for anteroposterior and lateral radiographs and discharged 2 to 3 days after surgery. X-Ray film of the injured vertebra was reviewed periodically after surgery. Nonsteroidal anti-inflammatory drugs (NSAIDs) or opiate analgesics cannot be given to patients after the vertebroplasty procedure, unless patients did not experience adequate pain relief.

### 2.4. Grouping Method

In this study, the VAS score was used to assess the mean severity of back pain in patients with OVCFs at 24 h, 3 d, and one month after PVP. Residual back pain was defined as the VAS score ≥ 4 both 3 d postoperatively and one month postoperatively. Finally, the patients were divided into group N and group R, based on the VSA score.

### 2.5. Evaluation Method

**Figure 2:**
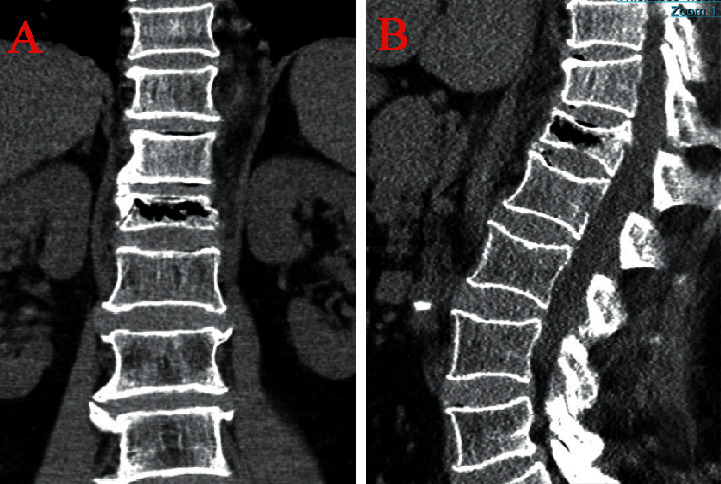
Intravertebral vacuum cleft was visible on coronal (a) and sagittal (b) views of computed tomography.

Pre- and postoperative factors that may affect back pain relief were evaluated, including the (1) demographic characteristics (gender, nationality, age, comorbidities (diabetes history, hypertension history, and fracture history), augmentation position, and BMI) and BMD; (2) VAS, ODI, and preoperative radiological parameters (AVH, AVHR, Cobb angle, intravertebral vacuum cleft (IVC) (Figure 2), posterior fascia oedema, and paraspinal muscle degeneration); (3) postoperative radiological parameters (volume, leakage, distribution of bone cement, AVHRR, and Cobb angle change).

**Figure 3:**
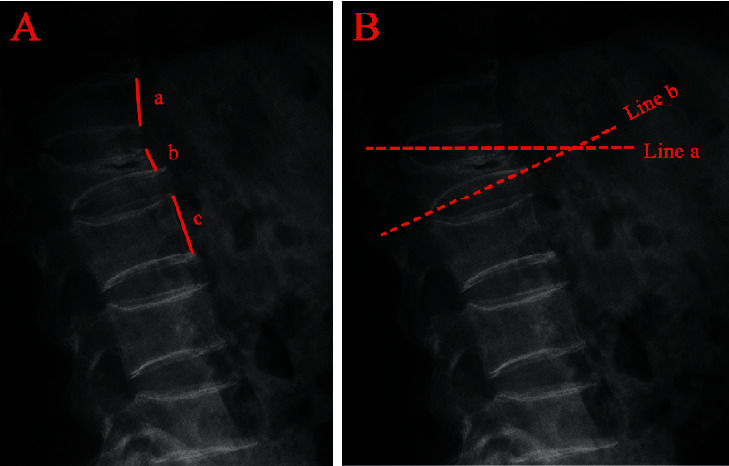
Radiographic evaluation of compressed vertebrae. (a) The anterior vertebral height ratio (AVHR) was defined as the height of the anterior wall of the compressed vertebral body (B)/(the height of the anterior wall of the upper vertebral body (A) + the height of the anterior wall of the lower vertebral body (C)) × 2. (b) Cobb angle was defined as the angle formed by the upper endplates (line a) and lower endplates (line b) of the fractured vertebral body.

**Figure 4:**
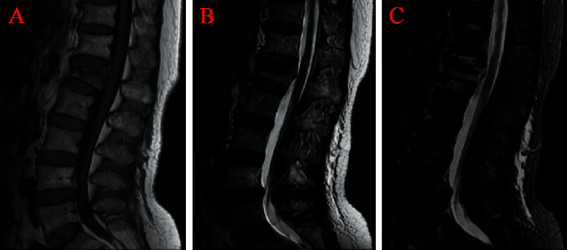
Posterior fascia oedema of osteoporotic vertebral compression fractures was visible on (a) T1 WI, (b) T2 WI, and (c) T2-STIR WI of MRI.

**Figure 5:**
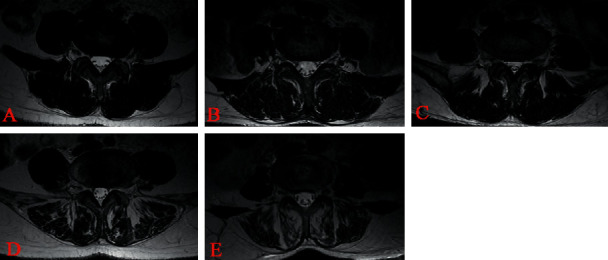
Goutallier grades 0 to 4 on T1W axial MRIs. (a) Grade 0, normal, almost no fat infiltration. (b) Grade 1, fatty streaks within the muscle. (c) Grade 2, fat infiltration was less than muscle mass. (d) Grade 3, fat infiltration was approximately equal to muscle mass. (e) Grade 4, fat infiltration was greater than muscle mass. Two experienced spine surgeons independently applied the Goutallier grades to the paraspinal muscle. Surgeons graded the paraspinal muscle for fat content at L4/5 levels on T1W MR axial images.

**Figure 6:**
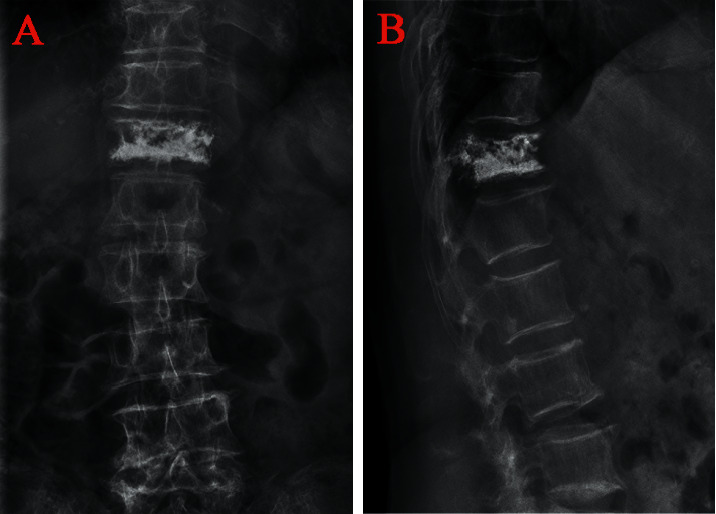
Distribution characteristics of blocky bone cement. (a) Anteroposterior X-ray film of local solid distribution pattern in the blocky group. (b) Lateral X-ray film of local solid distribution pattern in the blocky group.

**Figure 7:**
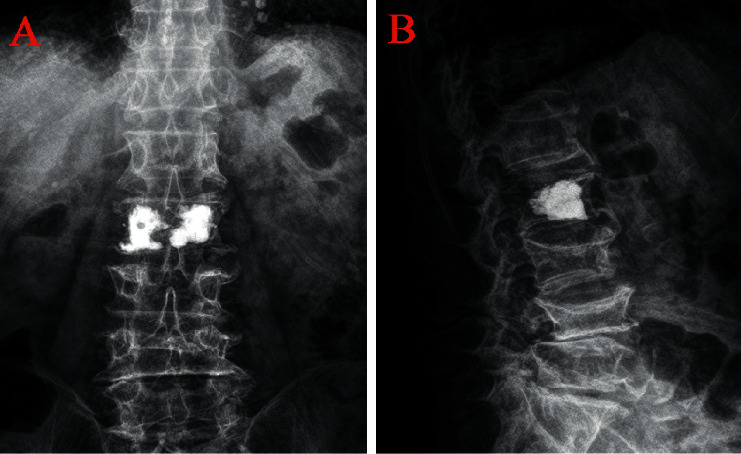
Distribution characteristics of spongy bone cement. (a) Anteroposterior X-ray film of diffuse distribution pattern in the spongy group. (b) Lateral X-ray film of diffuse distribution pattern in the spongy group.

The anterior vertebral height (AVH) and Cobb angle (LKA and Cobb's method) of the fractured vertebral body were measured before surgery and 24 h after surgery. AVH change was defined as postoperative AVH-preoperative AVH. The anterior vertebral height ratio (AVHR) was defined as the height of the anterior wall of the compressed vertebral body/(the height of the anterior wall of the upper vertebral body + the height of the anterior wall of the lower vertebral body) × 2 (Figure 3(a)). The anterior vertebral height recovery ratio (AVHRR) was defined as postoperative AVHR-preoperative AVHR. Cobb angle was defined as the angle formed by the upper and lower endplates of the fractured vertebral body (Figure 3(b)). Cobb angle change was defined as preoperative Cobb angle-postoperative Cobb angle. Posterior fascia oedema is defined based on the MRI finding (Figures 4(a)–4(c)). Paraspinal muscle degeneration is divided into 0-1, 2, and 3-4 grade bases on Goutallier grade classification [12] (Figures 5(a)–5(e)). Distribution of bone cement is divided into blocky and spongy [13] (Figures 6 and 7).

### 2.6. Statistical Analysis

Categorical variables were expressed as rates, and the chi-square test was used for comparison between groups. Continuous variables were expressed as mean ± standard deviation, and independent samples *t*-test or analysis of variance (ANOVA) was used for comparison between groups. Independent risk factors associated with residual back pain were assessed using multivariate logistic regression analysis.

A receiver operating characteristic (ROC) curve was drawn. The area under the curve (AUC) value was calculated to evaluate the sensitivity and the specificity. Nomogram was created by R software. Finally, we evaluated the stability of the prognostic nomogram by internal validation with 1000 bootstrap samples. Calibration plots were generated to examine the performance characteristics of the predictive nomogram. The Harrell's concordance index (C-index) was used to assess the prognostic accuracy. Decision curve analysis (DCA) was assessed whether the model improves forecasted net income. The data were statistically analyzed using SPSS version 24 (IBM Corporation, Armonk, NY, USA) and R version 3.6.1 (R Foundation for Statistical Computing, Vienna, Austria). Differences were defined as statistically significant at *P* < 0.05.

## 3. Results

Among 268 patients treated with PVP, 37 (13.81%) patients were classified postoperative residual back pain. Another 231 patients who were identified as having no residual back pain during the same period were included as group N. As shown in Table 1, although patients in the two groups were presented with back pain relief during the follow-up, the VAS score of the group R was significantly higher than that of the group N at 24 h, 3 d, and 1 month after surgery. The differences in demographic characteristics (gender, nationality, age, comorbidities (diabetes history, hypertension history, and fracture history), augmentation position, and BMI), and BMD between group N and group R were not statistically significant (Table 2).

The differences in VAS, ODI, AVH, AVHR, and Cobb angle between the two groups were not statistically significant (Table 3). There were 20 cases (7.46%) of IVC, 6 cases (16.2%) in group R, and 14 cases (6.1%) in group N, and the difference was statistically significant (Table 3). The incidence of preoperative posterior fascia oedema and paraspinal muscle degeneration (Goutallier grade 3-4) in group R was significantly higher than that in group N, and the difference was statistically significant (Table 3).

The volume of bone cement, AVHRR, and Cobb angle change were higher in group N than in group R, and the incidence of bone cement leakage was higher in group R than in group N, but none of the differences were statistically significant (Table 4). The incidence of blocky distribution of bone cement was significantly higher in group R than in group N, and the differences were statistically significant (Table 4).

The results of the multivariate logistical regression analysis showed that the presence of an IVC (OR 3.790, *P*=0.026), posterior fascia oedema (OR 3.965, *P*=0.022), server paraspinal muscle degeneration (OR 5.804, *P*=0.01; OR 13.767, *P* < 0.001), and blocky cement distribution (OR 2.225, *P*=0.041) were independent risk factors for residual back pain after PVP, as shown in Table 5.

**Figure 8:**
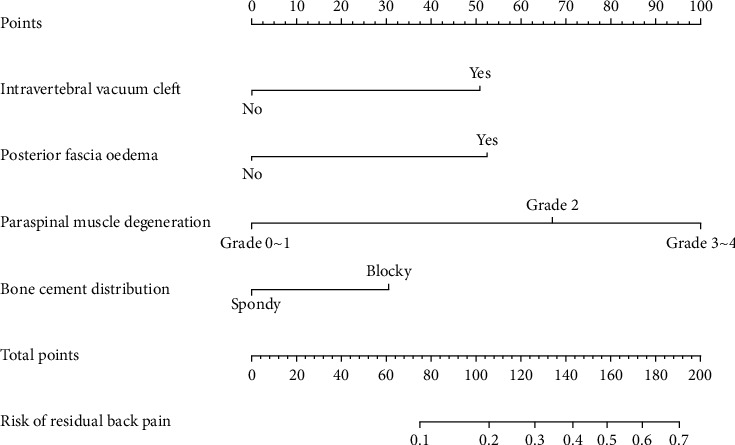
Predictive nomogram for residual back pain after percutaneous vertebroplasty.

**Figure 9:**
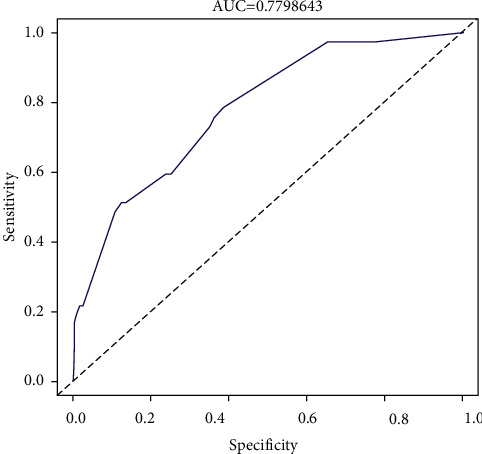
ROC curves for validating the discrimination of the nomogram prediction model.

**Figure 10:**
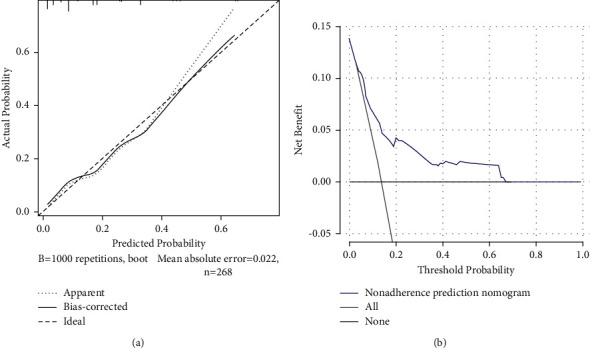
Calibration and decision curve of the nomogram for the probability of residual back pain after percutaneous vertebroplasty. (a) Calibration curve. (b) Decision curve.

To predict the risk of the residual back pain after PVP, we constructed a nomogram including the four independent risk factors based on the multivariate logistic regression results (Figure 8). To use the nomogram, a vertical line is drawn up to the top point row to obtain points for each variable, and then the sum of the points was calculated as the total score, and the predicted risk corresponding to the total score was the probability of residual back pain. The ROC of the prediction formula is presented in Figure 9. The AUC value was 0.7799, suggesting that the predictive ability was excellent. The calibration curve of the nomogram for the prediction of the residual back pain is presented in Figure 10(a), which demonstrated a good consistency between the probabilities predicted by the nomogram and the actual probabilities. The prediction nomogram presented good discrimination, with a C-index of 0.774 (0.696∼0.852), and was validated to be 0.752 through bootstrapping validation. Decision curves analysis used to assess the net benefit of the nomogram is illustrated in Figure 10(b). The result indicated that the nomogram was applicable when the threshold was in the range of 0.06 to 0.66 due to the net benefit.

## 4. Discussion

Percutaneous vertebroplasty is one of the most commonly used methods for the treatment of osteoporotic vertebral compression fractures, but there are still complications, including cement leakage, adjacent vertebral fractures, and inadequate postoperative pain relief [5]. Postoperative residual back pain greatly decreased patient satisfaction with surgery and significantly affected the quality of the patients' daily life [8].

Several studies have reported the postoperative residual back pain after PVP. Barr et al. performed a retrospective analysis of 38 patients with 70 vertebrae treated with PVP and reported significant pain relief in 24 patients (63%), moderate pain relief in 12 patients (32%), and inadequate pain relief in 2 patients (5%). Dohm et al. [14] compared PVP and PKP for osteoporotic vertebral compression fractures, reporting procedural pain (12/191 vs. 9/190) and back pain (14/191 vs. 28/190) as the most common adverse events at 30 days postoperatively. We found that the incidence of residual back pain at 30 days after PVP was 13.81% (37/268), which is in line with the results of past findings [14–16]. We selected one month as our postoperation study period, which aims to reduce the variation in risk factors associated with residual back pain due to the follow-up time varies. Multiple previous studies have shown that low bone mineral density, bone cement volume, bone cement distribution, preoperative posterior fascia oedema, and IVC may be associated with residual back pain after PVP [6,9,17,18]. In contrast, our findings found that bone cement distribution, preoperative posterior fascia oedema, and IVC were independent risk factors for residual back pain after PVP, which was consistent with those reported in previous studies.

In our study, we found that bone mineral density and bone cement volume were not risk factors for residual back pain after PVP. Osteoporosis is an important factor in the occurrence of vertebral fractures, as well as a risk factor for the collapse of fractured vertebral bodies after PVP and even secondary fractures of adjacent vertebral bodies. When multiple vertebral bodies collapse after surgery, the sagittal balance of the patient changes and the body compensates for the increased damage to the low back muscles in order to regulate the balance, leading to chronic low back pain, and the same is true for adjacent vertebral body fractures secondary to PVP [19,20]. Therefore, low bone density is an important risk factor for residual back pain after PVP. However, the results of this study found that the preoperative bone mineral density was lower than −2.5 SD in both groups, but the difference was not statistically significant. Analysis of the reason may be related to our time point of 1 month after PVP surgery. Collapse of the fractured vertebrae was not significant at the observation point of 1 month postoperatively and was much less likely to occur consecutively in multiple vertebrae. An important factor contributing to residual back pain after PVP is primarily sagittal imbalance, and patients do not experience significant sagittal imbalance 1 month after PVP [6,21]. Therefore, low bone density should probably be a risk factor for long-term residual back pain after PVP, rather than short-term (1 month postoperatively).

The injection of bone cement can enhance the vertebral strength and stiffness and effectively prevent the progressive vertebral collapse, thus relieving the back pain to some extent [22,23]. However, related studies have shown that there is no significant correlation between the amount of bone cement injected and the degree of pain relief [18,24,25]. The possible reason is that small doses of bone cement are already enough to enhance the strength of fractured vertebrae [25]. A biomechanical study has found that stiffness of the fractured vertebrae can be restored when the volume of bone cement reaches approximately 15% of the vertebral body [26]. Few significant benefits have been shown when the volume of cement used exceeds 24% of the vertebral body, at which point effective pain relief is already achieved [27,28]. The results of some studies have shown that satisfactory pain relief can be achieved by 1.5 ml bone cement [24,25]. The results of the present study are consistent with the results of previous studies that the amount of bone cement is not a risk factor for residual back pain after PVP. The possible reason for this is that the average bone cement injection volume in this study was greater than 3 ml, which is already the minimum dose for pain relief. We found that the rate of blocky bone cement distribution was higher in group R than group N, and multivariate logistic regression analysis showed that blocky cement distribution was an independent risk predictor of residual back pain after PVP. The spongy bone cement distribution can achieve tight binding between bone cement and cancellous bone, effectively reducing the risk of postoperative recollapse of the injured vertebra [29]. Meanwhile, more adequate bone cement distribution can effectively fix the fracture fragments, thus better relieving back pain [30].

Although the majority of patients with OVCFs are due to low-energy injury, there are still 18 patients with posterior fascia oedema. A prospective observational study by Yan et al. [9] found that the VAS and ODI scores were significantly lower in patients without posterior fascia oedema than in those with posterior fascia oedema, and a previous retrospective study has confirmed the correlation between posterior fascia oedema and residual back pain after PVP [6,7]. The results of our study are consistent with the above study that the posterior fascia oedema is an important risk factor for residual back pain after PVP.

PVP and PKP are considered the ideal methods to treat OVCFs with IVC [31]. However, some studies still report that the presence of preoperative IVC during long-term follow-up will more or less affect the clinical outcome after PVP, such as vertebral body recollapse, severe over residual back pain, or even compression fractures of adjacent vertebrae [13,32]. The presence of IVC sign in OVCFs has been reported to be a major risk factor for postoperative recollapse of the injured vertebra, progressive kyphosis, and chronic back pain [31,33]. Some scholars have analyzed the possible reason for this is an insufficient amount of bone cement filling the fibrocartilage membrane around the IVC, which prevents a tight bonding between the cement and the surrounding cancellous bone, thus causing instability of the fractured vertebrae [33,34]. Consistent with these studies, our results show that preoperative IVC is a risk factor for residual back pain after PVP.

Paraspinal muscle degeneration was not mentioned in previous studies as a risk factor for residual pain after PVP. However, the relationship between the paraspinal muscles and degenerative disc disease has become of great interest in recent years [11,35]. Several studies have found muscle atrophy of the paraspinal muscles in patients with lumbar spinal stenosis who have low back pain [36,37]. Meanwhile, strengthening functional exercises of the back muscles has been shown to be effective in reducing nonspecific pain [38]. Therefore, it can be speculated that there is a certain connection between paraspinal muscle degeneration and low back pain. The results of our study showed that the degree of paraspinal muscle degeneration was significantly higher in group R than group N, and multivariate regression analysis proved that paraspinal muscle degeneration was an independent risk factor for postoperative residual back pain. However, the Goutallier grade classification method was used to qualitatively assess paraspinal muscle degeneration in this study, and a more precise quantitative analysis of paraspinal muscle fat infiltration was lacking [39]. Therefore, quantitative analysis of paraspinal muscle fat infiltration is needed in the future and may be more helpful in clarifying the risk factors for residual back pain after PVP. Nomogram is a graphical model in which the probability of the outcome can be calculated. It has been improved to be a feasible model in risk prediction. So, we depicted and validated the nomogram based on postoperative imaging parameters. Our nomogram could provide a precise predictionability for the residual back pain with a C-index of 0.774 (0.696∼0.852) and was validated to be 0.752 through bootstrapping validation.

This study currently has some limitations. First, our study is a retrospective study with a relatively small sample size, which may result in some bias. Second, only PVP performed by bilateral approach has been taken into consideration, which may have introduced selection bias. However, previous studies demonstrated the PVP surgery approach (unilateral or bilateral approach) was not a significant influence factor for residual back pain [7]. Therefore, even if PVP performed by unilateral approach is included, the conclusion is not affected significantly. Third, we selected one month as our postoperation study period, the follow-up period was too short, therefore it was difficult to identify more risk factors, including BMD values, infection, recollapse of the injured vertebra, and adjacent vertebral body fractures. Thus, multicenter, large sample, and long follow-up period studies are needed to further establish the risk factors for residual back pain after PVP in patients with OVCFs. What's more, our nomogram had a good performance in internal validation, but it still needs to be assessed externally in wider populations.

## 5. Conclusions

Our findings suggest that the IVC sign, posterior fascia oedema, blocky cement distribution, and severe paraspinal muscle degeneration are important risk factors for residual back pain after PVP in patients with OVCFs. We provide a nomogram that accurately predicts the risk of residual back pain after PVP.

## Figures and Tables

**Table 1:** Summary of VAS after surgery between group N and group R.

Follow-up	Group N (*n* = 231)	Group R (*n* = 37)	*t*	*P* value
24 h	3.63 ± 0.48	5.16 ± 0.65	−16.997	≤0.001
3 d	2.66 ± 0.47	4.57 ± 0.65	−17.183	≤0.001
1 month	2.13 ± 0.65	4.24 ± 0.64	−18.318	≤0.001

**Table 2:** Comparisons of baseline between patients between group N and group R.

Parameter	Group N (*n* = 231)	Group R (*n* = 37)	*t*/*χ*^2^	*P* value
Gender				
Male	46 (19.9%)	7 (18.9%)	0.02	0.888
Female	185 (80.1%)	30 (81.1%)		
Nationality				
Han	204 (88.3%)	34 (91.9%)	0.411	0.521
Hui	27 (11.7%)	3 (8.1%)		
Age (years)				
<60	12 (5.2%)	2 (5.4%)	0.029	0.999
60∼70	86 (37.2%)	14 (37.8%)		
70∼80	93 (40.3%)	15 (40.5%)		
>80	40 (17.3%)	6 (16.2%)		
Diabetes history (%)				
No (cases)	205 (88.7%)	35 (94.6%)	1.167	0.28
Yes (cases)	26 (11.3%)	2 (5.4%)		
Hypertension history (%)				
No (cases)	121 (52.4%)	20 (54.1%)	0.036	0.85
Yes (cases)	110 (47.6%)	17 (45.9%)		
Fracture history(%)				
No (cases)	198 (85.7%)	32 (86.5%)	0.016	0.901
Yes (cases)	33 (14.3%)	5 (13.5%)		
Augmentation position				
T4–T10	21 (9.1%)	2 (5.4%)	0.552	0.759
T11-L2	114 (49.4%)	19 (51.4%)		
L3–L5	96 (41.6%)	16 (43.2%)		
BMI (kg/m^2^)	23.92 ± 3.61	23.66 ± 3.27	0.411	0.681
Bone mineral density (T score)	−3.27 ± 0.87	−3.32 ± 0.93	0.328	0.743

**Table 3:** Comparisons of VAS、ODI, and preoperative radiological parameters between patients between group N and group R.

Parameter	Group N (*n* = 231)	Group R (*n* = 37)	*t*/*χ*^2^	*P* value
VAS	6.41 ± 0.834	6.57 ± 0.647	−1.088	0.277
ODI	63.96 ± 11.35	64.51 ± 9.52	−0.283	0.778
AVH (mm)	15.12 ± 3.07	15.04 ± 2.96	0.155	0.877
AVHR (%)	49.82 ± 10.24	49.65 ± 10.13	0.095	0.924
Cobb angle (°)	27.34 ± 7.25	26.81 ± 8.26	0.403	0.687
IVC				
No (cases)	217 (93.9%)	31 (83.8%)	4.763	0.029
Yes (cases)	14 (6.1%)	6 (16.2%)		
Posterior fascia oedema				
No (cases)	219 (94.8%)	31 (83.8%)	6.183	0.013
Yes (cases)	12 (5.2%)	6 (16.2%)		
Paraspinal muscle degeneration				
Goutallier grade 0-1	92 (39.8%)	3 (8.1%)	24.83	≤0.001
Goutallier grade 2	94 (40.7%)	14 (37.8%)		
Goutallier grade 3-4	45 (19.5%)	20 (54.1%)		

**Table 4:** Comparisons of postoperative radiological parameters between patients between group N and group R.

Parameter	Group N (*n* = 231)	Group R (*n* = 37)	*t*/*χ*^2^	*P* value
Bone cement volume (mL)	4.11 ± 1.13	3.89 ± 1.01	1.087	0.278
Bone cement distribution				
Blocky	75 (32.5%)	21 (56.8%)	8.184	0.004
Spongy	156 (67.5%)	16 (43.2%)		
Bone cement leakage				
No (cases)	192 (83.1%)	29 (78.4%)	0.495	0.482
Yes (cases)	39 (16.9%)	8 (21.6%)		
AVHRR (%)	8.07 ± 2.68	7.58 ± 2.30	1.051	0.294
Cobb angle change (°)	6.15 ± 4.25	5.83 ± 3.16	0.547	0.586

**Table 5:** Multivariate logistic regression analysis for the influence factors of postoperative residual back pain after PVP.

	B	SE	Wald	*P* value	OR (95% CI)
IVC (yes)	1.332	0.598	4.973	0.026	3.790 (1.175∼12.227)
Posterior fascia oedema (yes)	1.337	0.602	5.232	0.022	3.965 (1.218∼12.907)
Paraspinal muscle degeneration			16.120	≤0.001	
Goutallier grade 2	1.758	0.681	6.661	0.010	5.804 (1.527∼22.064)
Goutallier grade 3-4	2.622	0.674	15.116	≤0.001	13.767 (3.671∼51.638)
Cement distribution (blocky)	−0.800	0.392	4.163	0.041	2.225 (1.032∼4.797)
Constant	−3.361	0.665	25.524	≤0.001	0.035

## Data Availability

Please contact the corresponding author for data requests.

## Abbreviations

OVCFs: Osteoporotic vertebral compression fractures
PVP: Percutaneous vertebroplasty
PKP: Percutaneous kyphoplasty
PMMA: Polymethyl methacrylate
DXA: Dual energy X-ray absorptiometry
MRI: Magnetic resonance imaging
BMD: Bone mineral density
BMI: Body mass index
VAS: Visual analog scale
AVH: Anterior vertebral height
AVHR: Anterior vertebral height ratio
AVHRR: Anterior vertebral height recovery ratio
IVC: Intravertebral vacuum cleft
ROC: Receiver operating characteristic curve
AUC: Area under curve
DCA: Decision curves analysis.
